# Tough challenges for testing Ebola therapeutics

**DOI:** 10.2471/BLT.15.020215

**Published:** 2015-02-01

## Abstract

Therapies for Ebola virus disease are urgently needed, but they must be rigorously tested for safety and efficacy before any mass roll-out to patients. Fiona Fleck reports.

At a briefing of United Nations officials on treating Ebola patients in western Africa with medicines and blood products in November last year, one question came up again and again: “Why is it taking so long?”

Officials wanted to know why Ebola patients who have been evacuated to Europe or North America have had higher survival rates than those who remained in the outbreak countries, says scientist Martin Friede, who leads the technology transfer team at the World Health Organization (WHO) in Geneva.

“Some of these patients had received a whole range of drugs – everything *and* the kitchen sink – but I explained to them that we don’t know what helped them to recover. Was it the clinical care? Was it the kitchen sink?” he says.

“That’s why we must do clinical trials to find out which drugs are safe and effective in these patients,” says Friede, a former vice-president of development at California biotech company Apovia Inc., who joined WHO in 2003.

“Was it the clinical care? Was it the kitchen sink?”

Martin Friede

Since WHO announced news of the Ebola outbreak in Guinea last March, the United Nations health agency has received more than 200 proposals of all kinds of therapies to treat Ebola virus disease suggestions.

Some suggestions – such as ingesting vulture gastric juices and plant root extracts or wearing magnets – were rejected for their lack of scientific evidence.

Others, including some drugs already licensed for other diseases, as well as novel drugs specifically aimed at Ebola that are under development, have been given to Ebola patients on “compassionate grounds”. So far, however, there are no definitive data available to suggest that these interventions are either effective or safe in Ebola patients.

Given the urgent need for additional therapies for Ebola – currently the only recommended management is replacement of fluids and electrolytes, and good control of symptoms – WHO is taking the lead in a major international drive to test potential therapies.

Since August, the UN agency has organized a series of meetings of experts to review the pipeline of potential therapies for Ebola virus disease.

As of 13 January, there were 21 373 cases and 8468 deaths in Guinea, Liberia and Sierra Leone, the three countries worst affected by the epidemic.

Past Ebola outbreaks were often small, confined to one community, and halted quickly by detecting and isolating cases, identifying contacts and safely burying the deceased – reasons why drug development for Ebola stalled in the past.

Clinical trials of potential therapies for Ebola can only be conducted during an outbreak, but there are enormous challenges with this.

**Figure:**
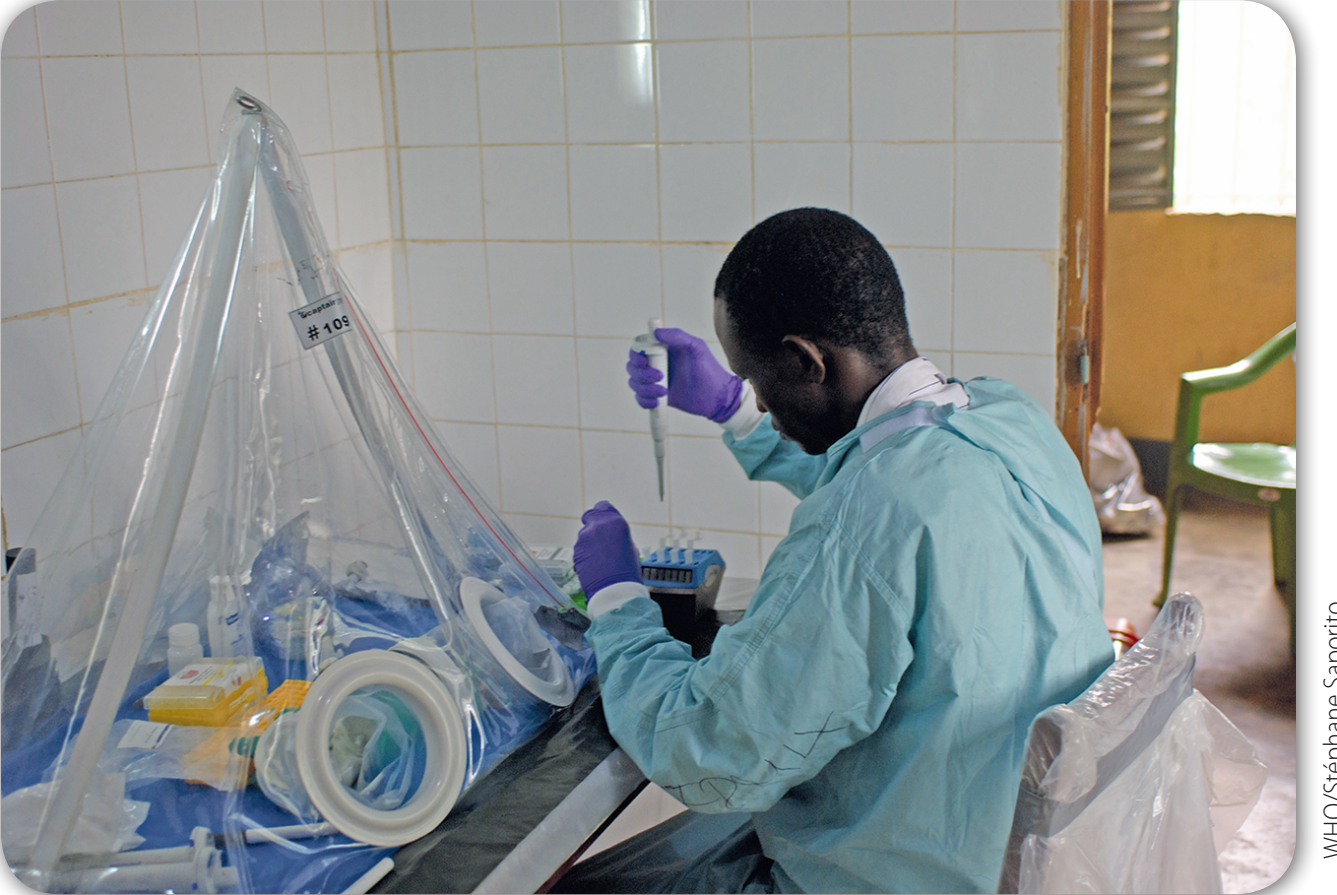
Laboratory worker tests samples for Ebola virus disease in Guékédou, Guinea, in 2014. Clinical trials of drugs or blood products for Ebola will rely heavily on well-functioning laboratories

WHO/Stéphane Saporito

“We identified only three products that work in the test tube and also give 100% protection in infected monkeys: ZMAPP (a cocktail of monoclonal antibodies), small inhibitory RNA, and antisense phosphorodiamidate morpholino oligomers, all targeting Ebola.

But we don’t know whether these are safe or effective in Ebola-infected patients and current supplies are non-existent or limited to quantities that are sufficient only to conduct very small clinical trials,” Friede says.

“So we drew up a short-list of repurposed drugs – i.e. ones developed for other conditions – including favipiravir, brincidofovir, toremefin and interferons, and we are continually reviewing this list as fresh data comes in on other drugs.

“With these repurposed drugs, there is less problem with supply, but a lack of clinical evidence of their effect against Ebola,” Friede says, adding that testing these drugs in animals infected with Ebola is hampered by the fact that they must be done in participating biosafety-level 4 laboratories, of which there are only a handful in the world. Each of these facilities can only handle a small number of animals at a time.

Favipiravir was developed by a Japanese company, Toyama Chemical, to treat influenza and some other viral infections and is being tested in Guinea for safety and efficacy in Ebola-infected humans by a team from the Institute of Health and Medical Research (INSERM) and Paris Diderot University in France. If favipiravir proves effective against Ebola, the Japanese government has offered to provide 1 million courses of treatment.

A trial started in Liberia last month [January] to test whether brincidofovir, an antiviral drug developed by US-based company Chimerix, improves survival in humans infected with Ebola virus, led by Professor Peter Horby at the University of Oxford.

“As brincidofovir is still being evaluated in animal studies, we have to continually re-evaluate our position – it’s a moving target,” Horby says.

A major issue for this and other potential Ebola therapeutics is ensuring availability and affordability of any drugs that prove effective.

“The drug will never be available unless you do the trials. But we do not want to wait until we have the data showing that it is effective before starting discussions with the drug companies and funders about production scale up and pricing,” Horby says.

According to a meeting of international ethics experts convened by WHO on 8 August 2014, if experimental drugs – i.e. those that have not been tested and licensed for use in humans to help them fight Ebola infection – are given to patients in the outbreak, “there is a moral obligation to collect and share all data generated”.

“Unfortunately this is not always taking place. Several interventions, including amiodarone, statins, antihypertensives and even intravenous ozone, have been tried by various medical teams,” Friede says. “Even where some data have been collected, they have not always been sufficient for a full assessment of the safety and efficacy of these approaches by WHO.”

“Reports on these ad-hoc tests, which have not gone through formal approval processes, have led to debate in social media about whether Africans were being used as human guinea pigs,” he says.

**Figure:**
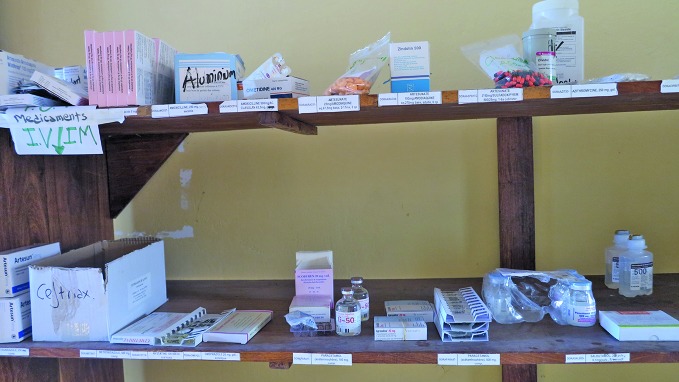
Medicines used to alleviate the symptoms of Ebola virus disease during the outbreak in Guinea, in 2014

WHO/Marie-Agnès Heine

There are also other concerns. All trials of Ebola therapeutics must be conducted under rigorous biosafety conditions, which means using full protective gear. The trials put researchers providing experimental blood transfusions and intravenous medicines at a risk of infection.

“The epidemic is moving in waves, and there are geographical hotspots,” Horby says, adding that some of the Ebola treatment units that were prepared as trial sites are no longer receiving enough patients. That could mean delays, if new sites are to be prepared, and would limit data collection.

“All these things make it pretty pressured to try and get these trials under way,” Horby says, recalling a call to action by a group of filovirus disease experts in 2007 to develop vaccines, therapies and diagnostics for Ebola in the* Journal of Infectious Diseases* (196:S136–41).

“There is the scientific urgency now and if we want to find an answer, we must do it during the epidemic. If we miss the epidemic, we will have failed again,” Horby says.

“If we miss the epidemic, we will have failed again.”

Peter Horby

While there is broad agreement about the potential of blood products to help Ebola patients recover, these remain untested therapies.

“The idea is to provide either plasma or convalescent blood to patients who are a blood group match for these products as doses come available, and since some people will not have a match, there will be no sense that treatment is being withheld,” says David Wood, who leads WHO’s vaccine regulation team.

“The trial design is to compare outcomes in the people receiving this treatment (the cases) with Ebola patients who do not have a matched unit of blood or plasma but receive standard supportive care (the controls),” he says.

“Plasma must be separated from convalescent blood. In Liberia and Guinea, this is being done at a mobile unit donated and funded by the Bill & Melinda Gates Foundation. But the rate at which survivors will volunteer to donate is not known, which makes it difficult to predict how long it will take to conduct the trials,” Wood says.

“It’s an untested therapy: there may be no added value in plasma over normal blood – as blood transfusion may help patients with such infections. People agree that blood products can be beneficial, but do you need blood from survivors?” he says. 

For Horby, it is vital to get the trials under way as soon as possible, while ensuring the safety of patients and health-care workers. 

“In the past, during outbreaks of severe acute respiratory syndrome (SARS), avian influenza and pandemic influenza A (H1N1), we were too slow and the epidemics were so fast that they passed and we didn’t find out which drugs work in these infections. This time we have the opportunity to do things differently”.

